# Bone Scan Index predicts skeletal-related events in patients with metastatic breast cancer

**DOI:** 10.1186/s40064-016-2741-0

**Published:** 2016-07-16

**Authors:** Ai Idota, Masataka Sawaki, Akiyo Yoshimura, Masaya Hattori, Yoshitaka Inaba, Isao Oze, Toyone Kikumori, Yasuhiro Kodera, Hiroji Iwata

**Affiliations:** Department of Breast Oncology, Aichi Cancer Center Hospital, 1-1 Kanokoden, Chikusa-ku, Nagoya, 464-8681 Japan; Department of Transplantation and Endocrine Surgery, Nagoya University Graduate School of Medicine, Nagoya, Japan; Department of Diagnostic and Interventional Radiology, Aichi Cancer Center Hospital, Nagoya, Japan; Division of Epidemiology and Prevention, Aichi Cancer Center Research Institute, Nagoya, Japan; Department of Gastroenterological Surgery (Surgery II), Nagoya University Graduate School of Medicine, Nagoya, Japan

**Keywords:** Breast cancer, Bone metastases, Bone Scan Index, Skeletal-related events

## Abstract

**Background:**

Bone Scan Index (BSI) expresses tumor burden in bone as a percentage of total skeletal mass, but its significance for metastatic breast cancer patients is unknown. We investigated whether baseline BSI is associated with skeletal-related events (SREs) or survival and identified the cut-off BSI score for predicting SREs in metastatic breast cancer patients.

**Methods:**

We retrospectively reviewed 144 patients with bone metastatic breast cancer. Bone scan examinations were performed and BSI was calculated using the Bonenavi^®^ automated method. All patients received standard medical treatment for metastatic breast cancer. For bone metastasis prophylaxis, bisphosphonates were infused initially with analgesics as needed. We defined SRE as either bony, requiring intervention (surgery and/or radiotherapy) for pain or prevention of fracture, or spinal cord compression. The rates of SRE and overall survival (OS) were evaluated according to baseline BSI, and the cut-off score of BSI for predicting SRE in metastatic breast cancer patients was identified.

**Results:**

Thirty-three patients (25.6 %) had SREs. The median BSI was 1.08 % (inter-quartile range 0.50–3.23 %). To identify the cut-off BSI score for predicting SRE, we performed sensitivity analysis to check *P*-value at every 0.1 BSI interval (0.4–2.4) by multiple-variable proportional hazard analysis. A BSI cut-off point of 1.4 % showed the lowest *P* value. Patients with BSI scores ≥1.4 had a significantly higher rate of SRE than those with lower BSI (*P* = 0.022). However there was no significant difference in OS.

**Conclusion:**

BSI may predict SRE in patients with metastatic breast cancer. A high BSI value (≥1.4) at diagnosis of bone metastasis may be a predictor of SREs in bone metastatic breast cancer patients.

## Background

Breast cancer is known to be associated with a high incidence of bone metastasis (Coleman and Rubens 1987; Hamaoka et al. 2004; Koizumi et al. 2010a, b), which causes skeletal-related events (SREs), including pain, bone fractures, spinal cord compression and hypercalcemia, significantly impairing patients’ quality of life (Costa and Major 2009; Onishi et al. 2010; Sturge et al. 2011). Early detection of metastatic disease may therefore prevent these complications, offer a better chance to control the disease process and result in better survival and better quality of life (Koizumi et al. 2010a, b). For diagnosis of bone metastasis, bone scintigraphy has good sensitivity (Costelloe et al. 2009) and has been regarded as the first alternative imaging method capable of diagnosing asymptomatic bone metastasis because it is readily available and provides an entire skeletal visualization within a reasonable amount of time and at a reasonable cost (Liu et al. 2011). It is widely used for patients with carcinoma to detect skeletal metastasis despite advances in other modalities such as PET-CT and MRI, although routinely examination is not always recommended in breast cancer patients. It can help diagnose a number of conditions relating to bones, including cancer of the bone or cancers that have metastasized to bone, although its findings are frequently non-specific because the uptake of ^99m^Tc-methyl diphosphonate depends on the integrity of osteoblasts and on matrix activity. There is the problem of mixed breast cancer metastasis (lytic and sclerotic). Indeed, lytic lesions are underestimated by bone scan and less well visualized also by Bone Scan Index (BSI), which is less the case for cancer of the prostate, where the lesions are almost all sclerotic therefore with a scintigraphy hot spot appearance. In breast cancer, the lytic lesions can be having the pattern of discreetly uptake or normally uptake but rarely with hot spot appearance.

On a per patient basis, the pooled sensitivity estimates for bone scintigraphy is 87.0 % and the pooled specificity estimates is 88.1 % in detecting bone metastases in patients with breast cancer (Liu et al. 2011). In addition an appropriate approach for quantitative analysis is required.

BSI has been developed as a quantitative tool to improve the interpretability and clinical relevance of bone scanning, making it possible to show the bone metastatic tumor burden (Erdi et al. 1997; Imbriaco et al. 1998). BSI has recently been reported as a response indicator in patients with metastatic prostate cancer (Dennis et al. 2012), as well as a prognostic indicator (Sabbatini et al. 1999). However, few studies have examined the significance of BSI for metastatic breast cancer patients (Colombié et al. 2013; Iwase et al. 2014).

The aims of this study were twofold: first, to explore whether baseline BSI was associated with SRE or survival and second, to identify the cut-off score of BSI for predicting SRE in metastatic breast cancer patients.

## Patients and methods

We retrospectively reviewed all breast cancer patients’ data from Aichi Cancer Center Hospital’s database between 2002 and 2012. Patients comprised 144 individuals with bone metastatic breast cancer, who had undergone whole body bone scan examinations in our institution at diagnosis of bone metastasis. The definition of bone metastasis was done by imaging findings such as X-ray examination, CT or MRI scan as well as subjective symptoms and bone scan. Bone scan examinations were performed 3 h after intravenous injection of 740 MBq ^99m^Tc-methyl diphosphonate (FUJIFILM RI Pharma Co., Ltd., Tokyo, Japan), using nuclear medicine imaging procedures to detect radioisotope tracer uptake in the patient’s body (Infinia HE4^®,^ GE healthcare, Tokyo, Japan). A gamma camera equipped with a low-energy high-resolution parallel whole collimator was used at anterior and posterior view scan speeds of 15 cm/min (matrix 256 × 1024). Energy discrimination was provided by a 10 % window centered on the 140 keV of the Tc^99m^. BSI was calculated first by determining the percentage of each bone that is involved by the tracer in relationship to the total skeletal mass, as determined from reference man, which is using the Bonenavi^®^ automated method (FUJIFILM RI Pharma Co., Ltd., Tokyo, Japan). We retrospectively reviewed imaging data to analyze BSI without having any clinical information of the patient. If patients had taken bone scan examinations more than twice, we analyzed the image at the time of bone metastasis initially. We excluded the scan data if it is before or after three months from the day of diagnosis.

All patients received standard systemic therapy for metastatic breast cancer, including chemotherapy, hormone therapy, and anti-HER2 therapy. For prophylaxis against SRE, bisphosphonates or denosumab were infused initially with analgesics as needed. We defined SRE as either bony, requiring intervention (surgery and/or radiotherapy) for pain or prevention of fracture, or spinal cord compression, and recorded the date on which an SRE was first observed. Patients with an SRE before bone scan examination were excluded. SRE-free survival was recorded from the date of baseline bone scanning to the SRE date. Overall survival (OS) was also recorded from the baseline bone scan to death. Written informed consent was obtained from all patients and this study has been approved in our institution (approved number: 2015-1-058).

The intrinsic subtype of primary tumor was classified using immunohistochemical (IHC) staining of paraffin-embedded thin sections as follows: luminal: ER and/or PgR positive (stained proportion >10 %), HER2 negative; luminal-HER2: ER and/or PgR positive and HER2 positive; HER2: ER negative, PgR negative and HER2 positive; triple negative: ER negative PgR negative and HER2 negative. A diagnosis of HER2 positive cancer was based on the published guidelines (Wolff et al. 2007).

The purpose of this study was to evaluate the rate of SRE and OS according to the baseline BSI, and to identify the cut-off score of BSI for predicting SRE in metastatic breast cancer patients. We performed sensitivity analysis checking *P* values for SRE at intervals of 0.1 from 0.4 to 2.4 % using a Cox regression model. We used the BSI score with the lowest *P* values as the cut-off point. To estimate the distribution of SREs and survival data, Kaplan–Meier estimates were used together with the log-rank test. Multivariate analyses were conducted using the Cox proportional hazard model. Factors evaluated in the model included age, metastasis site other than bone, performance status, and intrinsic subtype as well as the cut-off point of BSI. *P* values less than 0.05 were considered statistically significant. All statistical analyses were conducted using STATA^®^ v.12.1 (StataCorp, College Station, TX, USA).

## Results

Of the 144 patients who underwent a bone scan at metastatic diagnosis, 15 were found to have had SREs before the bone scanning date and were excluded, leaving 129 patients who were enrolled in the study. Patient characteristics are shown in Table 1. The median age was 57 years old (range 31–84) and ECOG performance status (PS) were 0–2, which are listed in Table 1. The proportion of each intrinsic subtype was as follows: luminal type; 66.2 % (n = 85), luminal HER2 type; 18.6 % (n = 24), HER2 type; 7.6 % (n = 10) and TN type; 7.6 % (n = 10). Almost all of the patients (95.3 %) had been treated with zoledronic acid and/or denosumab while five (3.9 %) received other bisphosphonate drugs. Twenty-three patients (17.8 %) had other distant metastases at baseline. The median BSI was 1.08 % (inter quartile range 0.50–3.23 %). The median follow up time for SRE was 2.04 years, and for OS was 2.50 years, respectively. During the clinical course, 33 patients (25.6 %) had SREs. Among them, 5 patients underwent surgical therapy and 28 patients were treated by irradiation.

**Table 1:** Patient characteristics (n = 129)

	N (%)
Age	
≥50	93 (72.1)
<50	36 (27.9)
Median age (range)	56 (31–84)
PS	
0	83 (64.3)
1	39 (30.2)
2	7 (5.4)
Intrinsic subtype	
Luminal	85 (66.2)
Luminal-HER2	24 (18.6)
HER2	10 (7.6)
Triple negative	10 (7.6)
Metastasis site with bone	
Brain	3 (2.3)
Liver	13 (10.1)
Lung	9 (7.0)
Zoledronic acid and/or denosumab use	121 (95.3)
Other bisphosphonate use	5 (3.9)
Median BSI (%)	1.08
Inter quartile range (%)	0.50–3.23
SRE	33 (25.6)
Surgical therapy received	5 (3.9)
Radiation therapy received	28 (21.7)

*PS* performance status

**Fig. 1:**
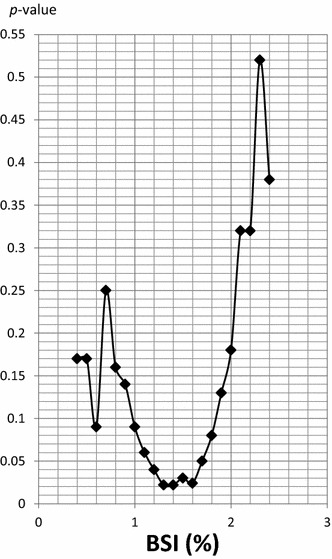
Sensitivity analysis for SRE by Cox regression model. A value of 1.4 for BSI showed the lowest *P* value in multivariate analysis

**Fig. 2:**
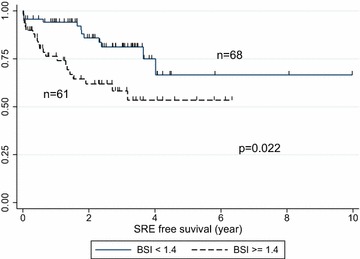
Kaplan–Meier curves for SRE (BSI cut off = 1.4). Patients with BSI ≥ 1.4 had significantly more SREs than those with BSI < 1.4 (*P* = 0.022). The median follow up time for SRE was 2.04 years

**Fig. 3:**
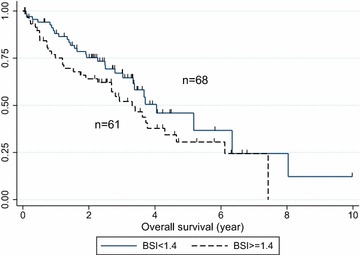
Kaplan–Meier curves for OS (BSI cut off = 1.4). It did not show any significance at that cutoff point (*P* = 0.436). The median follow up time for OS was 2.50 years

A BSI cut off-point of 1.4 % showed the lowest *P* value in multivariate analysis (Fig. 1). Twenty patients with higher BSI (≥1.4) and 13 patients with lower BSI (<1.4) had SREs. Figure 2 shows the Kaplan–Meier curves for SREs of the two groups. Patients with BSI ≥1.4 had significantly more SREs than those with BSI <1.4 (*P* = 0.022). The results of multivariate analysis for the risk of SREs are summarized in Table 2. Higher BSI (≥1.4) was a significant predictive factor (hazard ratio; HR 2.37; 95 % CI 1.13–4.95, *P* = 0.022), but other factors [age, metastasis organ, performance status (PS), intrinsic subtype] had no association with SRE. Kaplan-Meier analysis of OS in the two groups (Fig. 3) did not show any significance at that cutoff point (*P* = 0.436). Table 3 shows the association between OS and clinical factors including BSI. Higher BSI was not a prognostic factor for OS. The patients with PS2 or triple negative breast cancers had worse prognosis (HR 4.09; 95 % CI 1.41–11.9, *P* = 0.010, HR 2.94; 95 % CI 1.22–7.09, *P* = 0.016, respectively), but other factors were not statically significant.

**Table 2:** Multivariate analysis for SRE

	Hazard ratio	95 % CI		*P* value
BSI				
<1.4	1.0	Ref.		
≧1.4	2.37	1.13	4.95	0.022
Age				
<50	1.0	Ref.		
≧50	0.94	0.45	2.33	0.944
Metastasis site				
Bone only	1.0	Ref.		
Brain	N.E	–	–	–
Liver	1.35	0.34	5.41	0.675
Lung	0.99	0.22	4.53	0.990
PS				
0	1.0	Ref.		
1	1.06	0.47	2.40	0.890
2	2.030	0.45	9.10	0.354
Intrinsic subtype				
Luminal	1.0	Ref.		
Luminal HER2	1.52	0.65	3.55	0.330
HER2	0.98	0.22	4.44	0.980
Triple negative	0.58	0.76	4.57	0.612

**Table 3:** Multivariate analysis for OS

	Hazard ratio	95 % CI		*P* value
BSI				
<1.4	1.0	Ref.		
≧1.4	1.24	0.72	2.16	0.436
Age				
<50	1.0	Ref.		
≧50	0.91	0.50	1.66	0.768
Metastasis site				
Bone only	1.0	Ref.		
Brain	6.91	0.75	63.8	0.089
Liver	1.58	0.66	3.80	0.308
Lung	1.21	0.39	3.69	0.741
PS				
0	1.0	Ref.		
1	1.42	0.79	2.57	0.241
2	4.09	1.41	11.9	0.010
Intrinsic subtype				
Luminal	1.0	Ref.		
Luminal HER2	1.48	0.77	2.86	0.244
HER2	2.10	0.82	5.37	0.122
Triple negative	2.94	1.22	7.09	0.016

## Discussion

This study is the first to report the clinical significance of evaluating BSI when bone metastasis occurs in patients with metastatic breast cancer. Our results showed that the higher BSI group had a significantly higher rate of SRE than that of the lower BSI group. A BSI cut-off point of 1.4 % showed the lowest *P* value by multivariate analysis. At the cut-off point of 1.4 %, the higher BSI group had a significantly worse SRE rate than the lower BSI group. However, we found no statistically significant difference in OS.

BSI is a computer-assisted diagnosis system that eliminates differences between radiologists. The original BSI system was developed using a Swedish database (Sadik et al. 2008). Bonenavi^®^ is the Japanese version of EXINI bone^®^ (EXINI Diagnostics AB, Sweden), and is a commercially-available software package using a Japanese database (n = 904) (Horikoshi et al. 2012). The sensitivity of Bonenavi^®^ in diagnosing bone metastasis is 90 % and the specificity is 81 %, thus it is expected to be a better bone management tool (Horikoshi, et al. 2012). Recently this was revised to produce version 2, constructed from the database of 9 Japanese institutions (Nakajima et al. 2013). The feasibility of Bonenavi^®^ version 2 has been reported, and its accuracy is reportedly as good as version 1 (Koizumi et al. 2015).

In a study by Dennis et al. (2012) of patients with castration-resistant metastatic prostate cancer who received chemotherapy, BSI was found to be a response indicator. Indeed, they studied bone scans at baseline and at 3 and 6 months in different patients and BSI changes post-treatment were found to be a significant prognostic factor for survival (Dennis et al. 2012).

The results showed the feasibility of capturing bone scintigraphy data as a single quantitative measure and thereby allowing a bone scan to be explored for imaging biomarkers. Furthermore, changes in BSI post-treatment were significantly associated with survival, but post-treatment changes in PSA were not, while adjusting for changes in BSI. However the clinical impact of BSI in metastatic breast cancer patients is not yet known. One reason for the differences between castration-resistant metastatic prostate cancer and metastatic breast cancer may be that the mortality of metastatic breast cancer patients depends on control of metastasis to distant organs by chemotherapy or hormonal therapy.

In patients with metastatic breast cancer, therapy with bone-modifying agents is recommended to prevent the development of osteoporosis and SRE (Van Poznak et al. 2011). However extended exposure is associated with osteonecrosis of the jaw (Durie et al. 2005; Migliorati et al. 2006), so the timing of starting treatment with a bone-modifying agent is important. BSI may be helpful for selecting patients at high risk of SRE so that treatment with bone-modifying agents can be appropriately targeted, or choosing patients at low risk of SRE who are not necessary using bone-modifying agents.

The strength of this study is that it provides the first suggestion that BSI at diagnosis of bone metastasis is predictive for SRE. A limitation of this study is that treatment for metastatic breast cancer was heterogeneous because the study is retrospective. Brain metastasis had a trend as a prognostic factor for OS by the multivariate analysis, this is because only three patients were with brain metastases; it must be due to the weakness of the small number of this subgroup. There was also some variation in the length of time between the date of diagnosis of metastasis and the date of bone scan examination. As a next step, a prospective study to validate the clinical significance of BSI is needed.

## Conclusion

BSI may predict SREs in patients with metastatic breast cancer. The high cut-off level of BSI (≥1.4) at diagnosis of bone metastasis may be a predictor for SREs in bone metastatic breast cancer patients.
